# Dimensional Complexity of the Resting Brain in Healthy Aging, Using a Normalized MPSE

**DOI:** 10.3389/fnhum.2018.00451

**Published:** 2018-11-19

**Authors:** Norman Scheel, Eric Franke, Thomas F. Münte, Amir Madany Mamlouk

**Affiliations:** ^1^Institute for Neuro- and Bioinformatics, Universität zu Lübeck, Lübeck, Germany; ^2^Department of Neurology, Universität zu Lübeck, Lübeck, Germany; ^3^Department of Radiology, Cognitive Imaging Research Center, Michigan State University, East Lansing, MI, United States

**Keywords:** functional magnetic resonance imaging (fMRI), resting-state fMRI, Rockland sample, healthy aging, complexity, dimensional complexity, entropy, principal subspace analysis (PSA)

## Abstract

Spontaneous fluctuations of resting-state functional connectivity have been studied in many ways, but grasping the complexity of brain activity has been difficult. Dimensional complexity measures, which are based on Eigenvalue (EV) spectrum analyses (e.g., Ω entropy) have been successfully applied to EEG data, but have not been fully evaluated on functional MRI recordings, because only through the recent introduction of fast multiband fMRI sequences, feasable temporal resolutions are reached. Combining the Eigenspectrum normalization of Ω entropy and the scalable architecture of the so called Multivariate Principal Subspace Entropy (MPSE) leads to a new complexity measure, namely normalized MPSE (nMPSE). It allows functional brain complexity analyses at varying levels of EV energy, independent from global shifts in data variance. Especially the restriction of the EV spectrum to the first dimensions, carrying the most prominent data variance, can act as a filter to reveal the most discriminant factors of dependent variables. Here we look at the effects of healthy aging on the dimensional complexity of brain activity. We employ a large open access dataset, providing a great number of high quality fast multiband recordings. Using nMPSE on whole brain, regional, network and searchlight approaches, we were able to find many age related changes, i.e., in sensorimotoric and right inferior frontal brain regions. Our results implicate that research on dimensional complexity of functional MRI recordings promises to be a unique resource for understanding brain function and for the extraction of biomarkers.

## 1. Introduction

Resting-state functional magnetic resonance imaging (rs-fMRI) has become one of the main staples for understanding the functioning human brain (Biswal, 2012). When it comes to basic principles of brain function, the brain at rest, meaning in the absence of a dedicated task, seems to be a fruitful resource for brain state interpretations (Zhang and Raichle, 2010). Several studies demonstrated that, the resting brain is organized into independently acting, as well as interacting networks (Fox et al., 2005; Damoiseaux et al., 2006; Fox and Raichle, 2007; Smith et al., 2009; Binder, 2011; Shirer et al., 2012). Undoubtedly, the human brain is one of natures most complex information processing systems, but finding a measure to describe this complexity has been difficult (Sokunbi, 2016). Analyzing age-related changes in brain function is a nonetheless complex endeavor (Brodoehl et al., 2013).

Methods for studying the dimensional complexity of brain activity were already introduced for EEG-Data in the nineties, e.g., Ω-Entropy (Wackermann, 1996). As fMRI data quality and temporal resolution has increased significantly through multiband MRI sequences (temporal resolutions of whole brain recordings at a TR of ≈1s are easily achievable), these measures might now be of great value to understand the complex organization of brain function. Also with the help of online data sharing initiatives, like the 1,000 functional connectomes project^1^, access to vast amounts of high quality resting-state fMRI datasets has never been easier.

Predominantly, temporal patterns in rs-fMRI recordings are analyzed using linear correlation methods, averaging multiple voxel time courses to generate so called functional connectomes, neglecting non-linear temporal relations. This restriction to linearity might not be advised, as demonstrated by Pritchard et al. (2014). Using a non-linear information theory approach (cross-sample entropy) Pritchard et al. found the brain to be organized in “mega-hubs” with a scale-free degree distribution. In a review manuscript Sokunbi (2016) provides an overview on non-linear information theory based techniques, analyzing complexity of fMRI data in healthy aging, cognitive performance, Alzheimer's disease, attention deficit hyperactivity disorder (ADHD), schizophrenia and multiple sclerosis. Nevertheless, these approaches, based on Shannon entropy, operate on singular voxel (or averaged) time courses, some information theory approaches have extensions to two or multiple time courses but the immense computational demands to these measures make it almost impossible to get a holistic overview of brain states and brain complexity.

Here we want to introduce a new, whole brain complexity measure, analog to Ω-Entropy (Wackermann, 1996) but with adaptations to fit the needs of rs-fMRI. Like Ω, the normalized Multivariate Principal Subspace Entropy (nMPSE) uses the Eigenvalue spectrum of a subjects time and space data matrix. Contrary to Ω, nMPSE can be restricted in the amount of used EV energy, so that only the *k* dimensions with the highest variances are taken into consideration. Thus it is possible to reduce the state space to the most important dimensions. MPSE (not normalized), was shown to be efficient in differentiating between task and non-task periods in a task based fMRI study (Schütze et al., 2012). However, in a resting state scenario, due to the lack of normalization, MPSE is not independent of global data variance changes. Supplementary Figure 9 illustrates this effect on simulated data: While global data variance increases, MPSE also increases, nMPSE on the contrary, is not susceptible to this effect and stays constant even in the advent of global changes in data variance. Global variance is strongly influenced by multi-variate noise, at the foremost, subject motion. Even advanced artifact reduction methods like ICA-AROMA (Pruim et al., 2015) and multiband adapted physiological noise regression (Scheel et al., 2014a) are not able to fully eliminate this effect (Scheel et al., 2014b). In addition to physiological noise, global data variance is even more likely to modulate MPSE on real data, which would make any systemic interpretation very difficult. Thus, in this manuscript we will focus on normalized complexity measures, i.e., nMPSE and Ω, which are not predisposed to this effect.

With direct relation to independent component analysis (ICA) of spatial Resting-State-Networks (RSNs) and corresponding Temporal-Functional-Modes (TFMs) (Damoiseaux et al., 2006; Smith et al., 2012), dimensional complexity measures, such as nMPSE and Ω, aim to quantify the dynamics that drive functional brain networks: Assuming all networks are operating in approximately the same dynamic states, i.e., if energy is evenly distributed over all brain activity patterns, overall entropy is expected to be high. If there is a clear focus on one (or a few) networks, overall entropy of the system will be small. With measures as such, it would thus be possible to characterize the organization of temporal processes in the human brain.

Changes in brain organization due to healthy aging have been reported in several studies (e.g., Brodoehl et al., 2013; Beason-Held et al., 2016; Goldstone et al., 2016; La Corte et al., 2016; He et al., 2017; Peterson et al., 2017; Siman-Tov et al., 2017; Tremblay et al., 2017; Zhuang et al., 2017; Zhu et al., 2017). In accordance to Liu et al. (2013), Toussaint et al. (2014), and Sokunbi et al. (2015) non-linear brain complexity measures, derived from information theory, find changes in brain complexity as a result of healthy aging as well. This makes aging a perfect example to study holistic changes in brain complexity, as we expect dimensional complexity also to be affected by aging. With nMPSE applied to healthy aging, as a proof of concept, we want to demonstrate that dimensional complexity measures can be used to describe changes in brain organization, due to external factors. We want to advocate this unique way of looking at brain function, as a method that might give rise to biomarkers of neurodegenerative diseases, as well as a tool to describe basic brain function.

## 2. Material and methods

### 2.1. Nathan kline institute rockland sample

We used the so called Rockland sample (Nooner et al., 2012), a public dataset from the Nathan Kline Institute, that provides a community sample of a great number of subjects that underwent a multitude of scanning protocols. In the scope of this study we employed the multiband session with a spatial resolution of 2 × 2 × 2 mm, a TR of 1,400 ms and 404 volumes. All Rockland sample subjects from releases 1–4 (of now 10 releases), who were at least 18 years of age and had a maximum movement displacement of 3 mm were included. This lead to 186 viable subjects with a mean age of 46.6 (standard deviation = 18) years. The dataset also provides physiological recordings (heart rate and breathing), which were removed from the fMRI data using the multiband adapted version of RETROICOR-RVHR (Scheel et al., 2014a). Further preprocessing steps included head motion realignment (McFlirt, Jenkinson et al., 2002), brain extraction (BET, Smith, 2002) and 4mm isotropic smoothing (SUSAN, Smith and Brady, 1997), all from the FMRIB Software Library - FSL (Jenkinson et al., 2012). Remaining head motion induced artifacts were further reduced using ICA-AROMA (Pruim et al., 2015). All subsequent preprocessing steps were carried out using the Data Processing Assistant for Resting-State fMRI (DPARSFA) V4.3 (Chao-Gan and Yu-Feng, 2010). These steps consisted of cropping and reorienting the T1 anatomical volumes, co-registration of functional and anatomical volumes, functional volume normalization to MNI space using DARTEL (Ashburner, 2007), regression of nuisance covariates (linear trends, cerebro-spinal fluid and white matter signals) as well as temporal bandpass filtering from 0.01 to 0.08 Hz.

### 2.2. Dimensional complexity measures

A series of rs-fMRI volumes can be seen as an abstract geometrical representation of neurological states at discrete moments in time. This representation is known as the state space (Wackermann, 1996). It can be described by a matrix $X\;=\;\left[{\vec{x}}_{1},\ldots ,{\vec{x}}_{n}\right]\in {\mathbb{R}}^{d\text{x}n}$, where *d* equals the amount of data points for each discrete moment in time *n* of all *N* time points. Principal Component Analysis - PCA (Jolliffe, 2002) can be used to find an orthonormal system of base vectors, so that any data vector ${\vec{x}}_{n}\in X$ can be expressed as a linear combination of orthonormal base vectors. For this, PCA calculates the Eigenvectors ${\vec{v}}_{1},\ldots ,{\vec{v}}_{d-1}$ of the covariance matrix *C*, which is defined by

$$c_{ij}=\frac{1}{N}\overset{N}{\underset{n=1}{\sum }}\left({\vec{x}}_{in}-\bar{X}\right)\left({\vec{x}}_{jn}-\bar{X}\right),$$

where $\bar{X}$ is the mean of *X*. The magnitude of an Eigenvalue (EV) λ_*i*_ of the corresponding Eigenvector ${\vec{v}}_{i}$, correlates with the variance of the data in direction of ${\vec{v}}_{i}$ (Jolliffe, 2002). This leads to multiple boundaries: firstly the trivial (*n*−1) boundary, where all EVs greater than *n* = *min*[*dim*(*x*), *dim*(*y*)] are zero. By removing all $\vec{v}$ where the EVs are zero, a loss-less reduction and optimal projection of the original data, into a space of lower dimension can be achieved. The second threshold lies below the (*n*−1) boundary: here we define it at the point, where the summed energy of the EV spectrum reaches 99%. Depending on the EV distribution, it might be the case, that 99% of EV energy is reached with a fraction of all possible (*n*−1) EVs. The remaining dimensions, above this boundary, are usually interpreted as additive noise and discarded. Boundaries, set at even lower percentages of EV energy are probably the most interesting ones: they only use the first dimensions, carrying the most prominent data variance. While the 99% boundary can be seen as a noise filter, lower boundaries might give an interesting insight into the most discriminating dimensions.

The first measure of dimensional complexity we use, is Ω-Entropy (Wackermann, 1996):

$$\Omega \left(X\right)=2^{-\overset{d}{\underset{i=1}{\sum }}\lambda_{i}^{\prime }\cdot {\text{log}}_{2}\left(\lambda_{i}^{\prime }\right)}.$$

It uses the information of the proportions between **all** EVs (normalized to a unit sum):

$$\lambda_{i}^{\prime }=\frac{\lambda_{i}}{s},s=\overset{d}{\underset{i=1}{\sum }}\lambda_{i}.$$

A dimensional complexity measure, that allows scalable levels of EV-energy is called Multivariate Principal Subspace Entropy (MPSE) and was introduced by Schütze et al. (2012). With MPSE, it is possible to restrict the number of EVs, using a parameter *k*:

$${\text{MPSE}}_{k}\left(X\right)=\frac{1}{2}\overset{k}{\underset{i=1}{\sum }}\text{ln}\left(\lambda_{i}\right)+\frac{k}{2}\left(1+\text{ln}\left(2\pi \right)\right),$$

Using normalized Eigenvalues λ′ (as used with Ω) instead of λ adds a normalization step to the *k*-scalable MPSE and leads to the normalized Multivariate Principal Subspace Entropy (nMPSE):

$$\begin{array}{lll} {\text{nMPSE}}_{k}\left(X\right) & = & \frac{1}{2}\overset{k}{\underset{i=1}{\sum }}\text{ln}\left(\lambda_{i}^{\prime }\right)+\frac{k}{2}\left(1+\text{ln}\left(2\pi \right)\right),\\ \text{where}\lambda_{i}^{\prime }=\frac{\lambda_{i}}{s},\;s=\overset{k}{\underset{i=1}{\sum }}\lambda_{i}. \end{array}$$

Here only the *k* largest EV dimensions (normalized to a unit sum) are taken into consideration. Supplementary Figure 9 shows a simulation of Ω, MPSE and nMPSE, displaying the invariance of nMPSE and Ω to global shifts in data variance. A Matlab implementation of nMPSE can be found in the Supplementary Material.

### 2.3. Brain parcellation, complexity and aging

**Figure 1:**
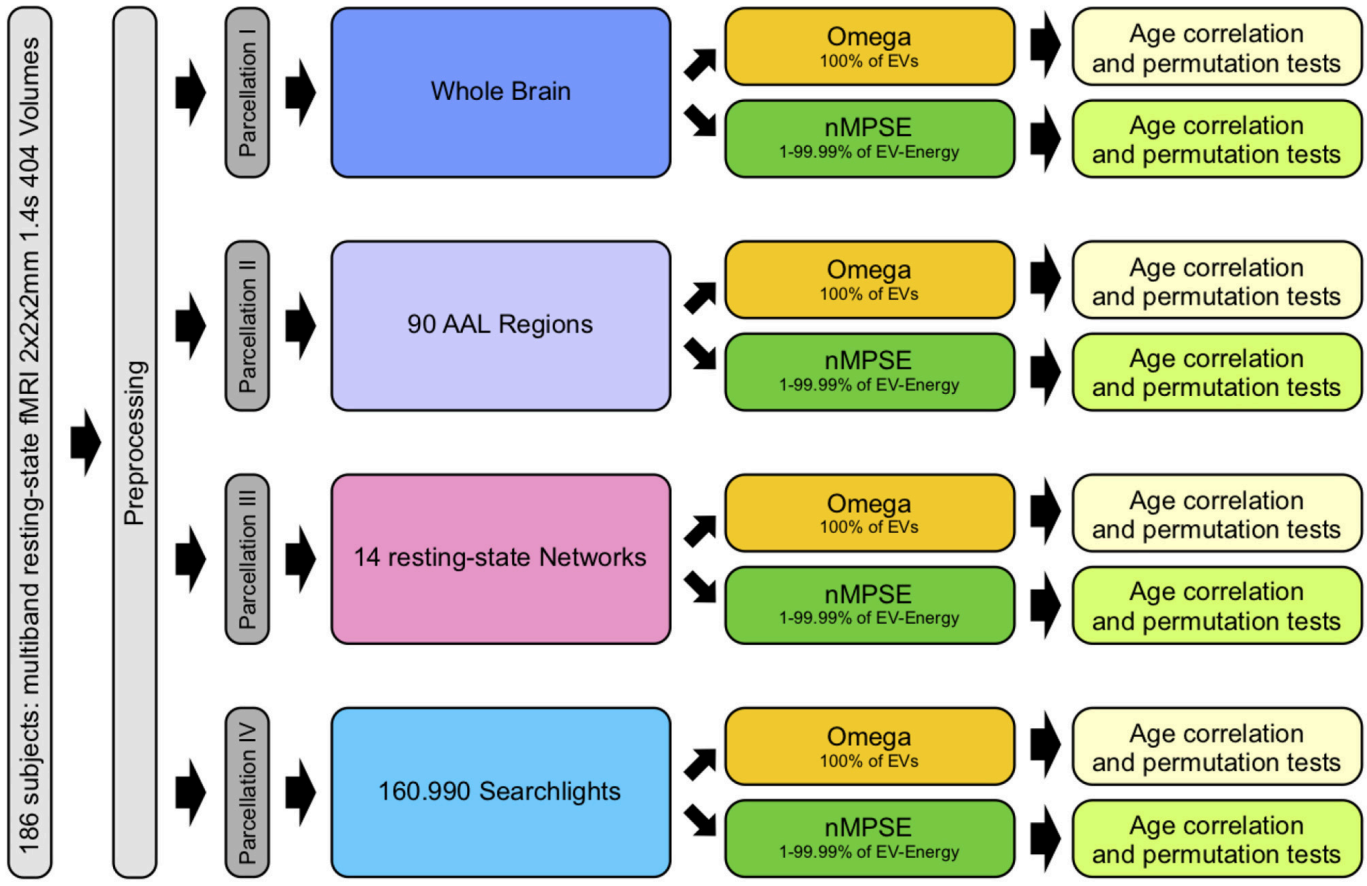
Workflow: after preprocessing, parcellation I was used for whole brain, II for anatomical regions, III for functional networks and IV for searchlight analyses. Each analysis consisted of calculating Ω (using all EVs) and nMPSE (using EVs that range from 1 to 99.99% of the summed EV-energy), followed by a correlation with corresponding subject ages and tests for significance.

In order to examine and study complexity of the aging brain, we used different brain parcellation approaches. The Automated Anatomical Labeling (AAL) atlas (Tzourio-Mazoyer et al., 2002) consists of 90 anatomical brain regions. Merging all 90 regions results in a full brain mask, subsuming all cortical brain regions. This mask served for whole brain analyses. The next approach examined complexity on each anatomical region separately. Here brain complexity was computed within each single AAL region. As a counterpart to anatomical parcellation, we also calculated complexity on functional network level by using the resting-state network atlas by Shirer et al. (2012). Here, we retrieved a complexity measure for each of the 14 resting-state networks. An even more fine-grained picture was achieved by employing a computationally demanding search light approach. For every voxel (search light) a sphere with a radius of 10 mm was created. This way we were able to calculate complexity within each of these spheres and assign a complexity value to every single voxel of the brain. Finally, to find a connection between brain complexity and biological age, we calculated the corresponding Pearson correlation of complexity and age for each parcellation approach. To evaluate statistical significance we used permutation tests and corrected for multiple testing using the Bonferroni method (Bonferroni, 1936). Figure 1 provides an overview of all different steps carried out.

## 3. Results

**Figure 2:**
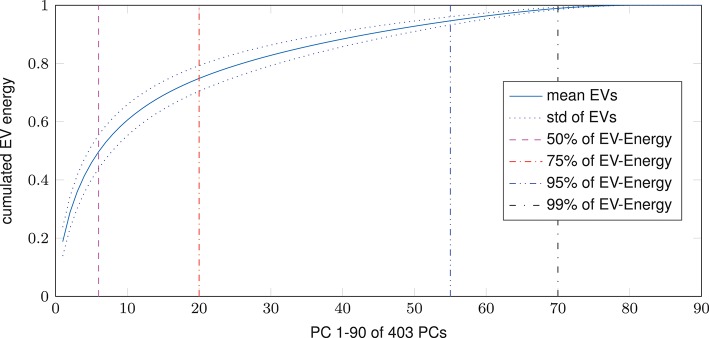
Cumulated EV spectrum, normalized to a unit sum, of all subjects (mean and standard deviation), showing multiple boundaries of the cumulated EV energy for 1–90 of 403 (*min*(160990 voxel, 404 time steps)-1) possible non-zero EVs resp. principal components - PCs.

Dimensional complexity measures, Ω and nMPSE alike, are based on a subjects Eigenvalue (EV) spectrum. Figure 2 displays the cumulated, whole brain, EV spectrum, normalized to a unit sum, showing the mean of all subjects with the corresponding standard deviation. As expected, 99% of Eigenenergy are reached using just a fraction of all possible non zero EV dimensions, namely 70 of 403. Already the first 6 dimensions make up for 50% of data variance, while 75% of Eigenenergy is represented in the first 20. This is in line with the expectation, that most of the data variance is dominated by resting-state network activity, as previous studies reported approximately 20 non-noise components, subsumming the activity of 14 resting-state networks (Shirer et al., 2012).

The next steps consist of analyzing the correlation of functional brain complexity and the subjects actual ages. Here, complexity is measured by either Ω (using the complete EV spectrum of each subject) or nMPSE (using *k*-thresholded EV spectra of each subject). In line with Figure 1, the following results are sectioned into the four different parcellation strategies: the analysis of whole brain functional complexity, followed by (hypothesis driven) anatomical regions, as well as functional resting-state networks, culminating in hypothesis free, whole-brain - small neighborhood search lights.

**Figure 3:**
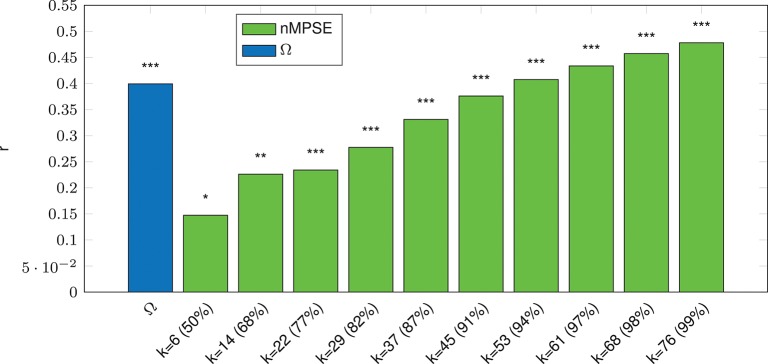
Whole-Brain complexity vs. age correlation for Ω and nMPSE (nMPSE labels show *k*-value and the corresponding percentage of the cumulated EV energy). Stars denote corrected significance levels with *p ≤ 0.05, **p ≤ 10^−2^, and ***p ≤ 10^−3^.

We can report a strong significant correlation for whole-brain Ω entropy and age (*p* ≤ 10^−3^, incl. Bonferroni correction), see Figure 3. Note that Ω uses all dimensions, meaning the complete EV spectrum to calculate the complexity. On the contrary, whole-brain nMPSE results show that 50% of EV energy (meaning using only the first six dimensions) already yield a significant, yet not strong correlation of nMPSE and age (*p* ≤ 0.05). Adding dimensions causes nMPSE-age correlation to increase. At roughly 91% of EV energy (45 components), nMPSE is on par with Ω, even surpassing it with more added EV energy. Using these observations, we can now try to identify EV energy bands, prone to age related changes and it turns out that the age effect is not restricted to some, or only the first few dimensions, as one would expect with 14 dominant resting-state networks. Apparently all meaningful whole brain components are affected by age related changes.

**Figure 4:**
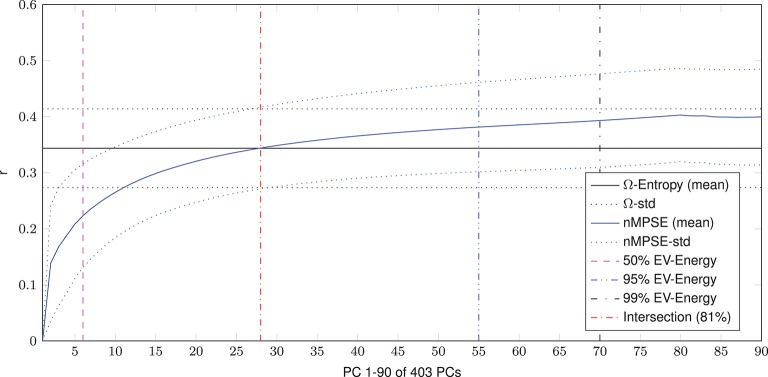
Mean age vs. complexity correlation for all anatomical regions. Using the first 50% of EV energy (the first 6 dimensions), nMPSE reaches significance. At this point the correlation for nMPSE is lower than for Ω. Adding dimensions, nMPSE surpasses Ω at 28 dimensions (81% of EV engery), the so called Ω-nMPSE intersection point. A dent at 82 dimensions represents the 99.99% EV energy boundary.

As global, whole brain, age effects are quite distributed over the EV spectrum, the question arises, if there is a spatial effect of different separate substructures of the brain. Looking at complexity within single anatomical regions, essentially replicates the finding of whole-brain effects (Figure 4). On average, Ω vs. age correlation is strongly significant (using the complete EV spectrum), while nMPSE vs. age correlation depends on the amount of EV energy used. The mean characteristic curve of nMPSE, subject to *k*, reaches Ω level at the intersection point of 81% of EV energy. Also, nMPSE significantly surpasses Ω, reaching *r* = 0.4 using 99% of EV energy. Again, we need a *k* higher than we would expect from Figure 2, to obtain an nMPSE-age correlation in the range of Ω vs. age.

**Figure 5:**
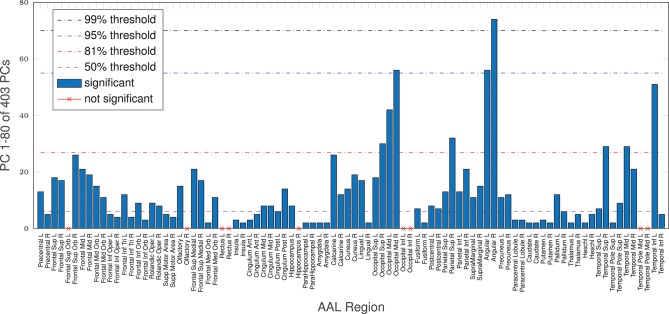
AAL regions, reaching a significant age/complexity correlation (*r* = 0.2, approximately) at diverse levels of EV energy. Some regions need extremely few dimensions, others need almost all EV energy available to reach significance. See Supplementary Table 3 for a listing of AAL regions with corresponding dimensionality results.

Additionally, even though the whole-brain effect is replicated on average with AAL regions, age-complexity correlations are regionally very diverse. Some regions show a strong significant complexity-age correlation, others do not. All in all, 81 (of 90) reach a significant complexity-age correlation using nMPSE, 71 using Ω. All 9 regions, not reaching significance with nMPSE, are a subset of the 19 regions not reaching significance with Ω. Furthermore, for regional nMPSE, very different amounts of EV energy are needed, see Figure 5. Some regions only demand very few dimensions, whereas others take up almost all of the EV energy to reach significance: e.g., the right insula or the right lingual AAL region only need the first two dimensions, corresponding to 29% of EV energy, in contrast, the right angular gyrus needs 74 components at 99% of EV energy. Most of the regions using less than the first 50% of EV energy, reach significance with only 2 or 3 dimensions. Supplementary Table 3 provides a lookup table for the results of each anatomical brain region.

**Figure 6:**
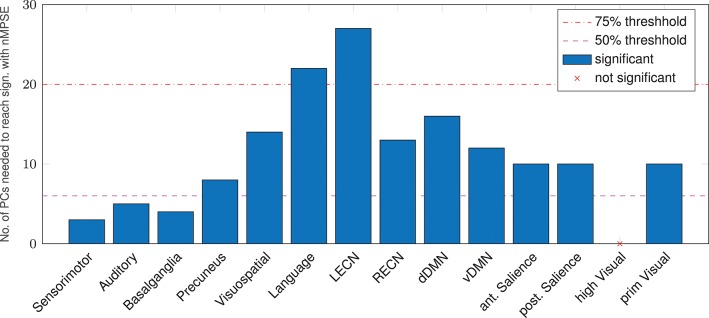
Resting-state networks, reaching a significant age/complexity correlation (*r* = 0.2 approximately) at diverse levels of EV energy. Some networks need extremely few dimensions (below the 50% threshold), others need quite a lot of EV energy. L/RECN marks the left/right executive control network, d/vDMN the dorsal/ventral Default Mode Network.

An alternative to anatomical brain parcellation is a functional approach, dividing the brain into functional rather than anatomical units, namely resting-state networks. Analyzing the age-effect from this point of view (Figure 6), we found that, as for anatomical regions, some networks are significantly correlated even for small *k*, others are more demanding. The Sensorimotor network needs the smallest number of dimensions to decode the age difference, whereas the left executive control network needs most of the EV energy to reach significance. Again, keep in mind that Ω uses the whole EV spectrum and reaches strong age correlations in all resting-state networks but one: Remarkably, corresponding to the AAL regions, the higher visual network did not show a significant correlation for Ω or nMPSE at any *k*.

**Figure 7:**
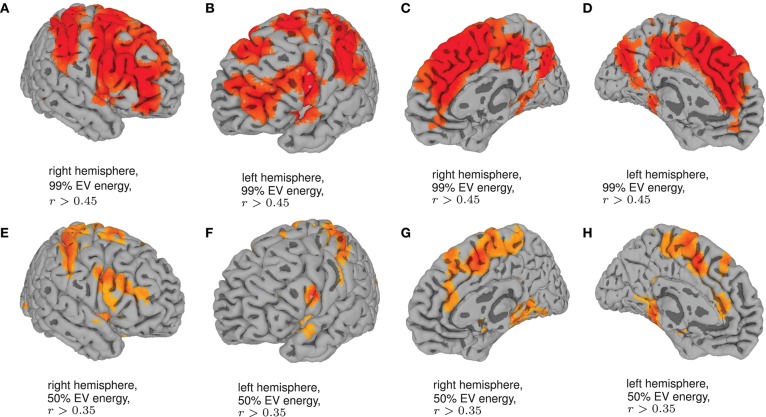
Searchlights for nMPSE *k* = 76 (99% of EV energy) and *k* = 6 (50% of EV energy). **(A)** Right hemisphere, 99% EV energy, *r* > 0.45. **(B)** Left hemisphere, 99% EV energy, *r* > 0.45. **(C)** Right hemisphere, 99% EV energy, *r* > 0.45. **(D)** left hemisphere, 99% EV energy, *r* > 0.45. **(E)** Right hemisphere, 50% EV energy, *r* > 0.35. **(F)** Left hemisphere, 50% EV energy, *r* > 0.35. **(G)** Right hemisphere, 50% EV energy, *r* > 0.35. **(H)** Left hemisphere, 50% EV energy, *r* > 0.35.

Finally, in order to have an hypothesis free grasp of brain regions, displaying complexity changes due to healthy aging, we look at whole brain, local neighborhood searchlights: Figure 7 shows the voxel populations yielding the highest age-complexity correlations, subject to the different levels of used EV energy. Figures 7A–D show, that using 99% of EV energy leads to wide spread, strong positive correlations between age and nMPSE. Table 1 lists the top ten voxel coordinates with corresponding locations and *r* values. On the contrary Figures 7E–H, using only 50% of EV energy, show a more concise view of aging effects, as only the first six dimensions were taken into consideration. Table 2 gives the corresponding list of top ten voxel coordinates. Results for Ω and intermediate nMPSE results (at different *k* levels) can be found in the Supplementary Material. Essentially, regional and network results are replicated by the searchlight approach. It seems like there is a filter-like transition from small *k* to big *k*: small regions grow naturally, while *k* increases. Small *k* regions appear to be seeds for larger *k* regions. Additionally some regions only appear when using higher *k* values (see frontal regions of left hemisphere).

**Table 1:** Top 10 significant voxels for nMPSE (*k* = 76, 99% of EV energy) searchlights.

***X***	***Y***	***Z***	***r***	**AAL Region**
48	18	18	0.6664	Frontal Inf Tri R
6	8	50	0.6272	Supp Motor Area R
−58	−32	48	0.619	Parietal Inf L
28	6	56	0.6179	Frontal Mid R
−58	12	18	0.6119	Frontal Inf Oper L
46	−36	58	0.601	Postcentral R
62	−24	42	0.5983	SupraMarginal R
−20	8	58	0.5766	Frontal Sup L
−8	−38	28	0.5663	Cingulum Post L
−30	32	20	0.5662	Frontal Mid L

**Table 2:** Top 10 significant voxels for nMPSE (*k* = 6, 50% of EV energy) searchlights.

***X***	***Y***	***Z***	***r***	**AAL Region**
10	8	56	0.5352	Supp Motor Area R
32	24	−12	0.5165	Frontal Inf Orb R
46	16	16	0.4924	Frontal Inf Oper R
−62	10	16	0.4852	Frontal Inf Oper L
−24	−40	−12	0.4814	Fusiform L
−6	8	0	0.4803	Caudate L
24	−40	64	0.4784	Postcentral R
−6	4	50	0.4732	Supp Motor Area L
18	−26	66	0.4718	Precentral R
−18	−24	64	0.4718	Paracentral Lobule L

## 4. Discussion

Looking at the results, we can see that dimensional complexity measures, describing the entropy of (sub-)spaces of a subjects Eigenvalue (EV) spectrum, can be successfully deployed on resting-state fMRI data, in order to find subtle differences in the organization and complexity of brain function. In addition to Ω for fMRI (initially introduced by Wackermann in 1996 for EEG analysis) we introduced and evaluated the normalized Multivariate Principal Subspace Entropy (nMPSE) for resting-state fMRI data. One of the main differences between Ω and nMPSE is the fact that, with nMPSE we can analyze entropy at different levels of *k*, i.e., for subsets of Eigenvectors (subspaces). This becomes especially interesting in the scope of resting-state networks, because we expect them to be related to the most variant principal components (PCs). Resting-state networks have been studied in many ways and have proven to be consistent across multiple data sets, as 10–20 components are reliably identified as parts of the 14 resting-state networks (Damoiseaux et al., 2006; Shirer et al., 2012). Looking at the averaged Eigenspectrum in Figure 2 we see a close resemblance to these findings: 20 components make up for 75% of cumulated EV energy, thus most of the data variance can be attributed to a comparable number of components.

As a proof of concept, we looked at the effects of healthy aging on dimensional complexity measures. In the following, we discuss the results of the whole brain functional complexity analysis. To further discern the contribution of substructures of the brain to the aging effect, we then discuss the results for functional networks as well as anatomical regions. The results of the regional hypothesis free, local neighborhood, whole brain searchlights will be discussed thereafter.

Whole brain functional complexity analysis (Figure 3) illustrates that, the first 10 dimensions, accounting for more than 50% of information, already include major age-related changes. The remaining dimensions cover the other half of the age-related effects. Routinely the analysis would be limited to 30 dimensions (approx. 85% of data variance) for comparable component based analyses. Doing so, barely leads to a stronger correlation. We found that nMPSE becomes more and more discriminative for a growing *k* at values even larger than 30. This indicates that *all* dimensions are changed by age-related processes, and not only the principal components with the largest Eigenvalues. Obviously there is a lot of discriminative information in higher dimensions with lower variance. In other words, the age-related correlation is not depending on just some prominent factors (e.g., single resting-state networks) but it seems like a globally distributed effect, that is independent of global variance (ruled out through normalization in Ω and nMPSE).

Not surprisingly, therefore all networks (except the higher visual network) showed a significant correlation between Ω and age. Using nMPSE, we could further examine the amount of EV energy needed to reach this significance. Here the higher visual network also never shows a significant correlation to age, which goes along with the observations using AAL regions. A closer look at Figure 6 reveals, that basic networks (i.e., sensorimotor, basal ganglia, and auditory), only need a small number of dimensions. Higher-order networks (i.e., executive control or language) need a lot more EV energy. One possible interpretation would be, that age-related changes for basic networks occur already in the first PCs of those networks, while higher order network changes along age, are wide-spread over many components. The dissent between the language network results here and respective areas from the AAL or search light based approach, can be explained by the fact, that the language network map is comprised of multiple language processing areas, while for the AAL and search light approach specific language related regions are tested separately.

We were able to replicate these findings quantitatively for all tested parcellation approaches. Specifically, using the AAL atlas demonstrated that, on average, we also need most of the components to retrieve a strong significant complexity-age correlation. Looking at AAL regions separately, we see that some yield significant correlations, using only the most dominant PCs (*k* ≤ 6). It seems, that areas naturally attributed to aging are also needing the least amount of EV energy to yield a significant result. Most interestingly areas responsible for motion control and motor processes, cognitive processing and decision making, memory encoding and retrieval, decision-making, emotions, language processing, inhibitory control and regulation of sleep as well as alertness (namely putamen, orbifrontal cortex, parahippocampal gyrus, amygdala, caudate nucleus and thalamus) are displaying the strongest correlations and are in line with other publications reporting aging effects in the brain (e.g., Brodoehl et al., 2013; Zhuang et al., 2017; Zhu et al., 2017). On the contrary, areas for processing visual information like calcarine, cuneus, angular gyrus among other occipital regions need almost all EV energy if they reach significance at all, hinting at an age independence, see Supplementary Table 3.

Employing the searchlight approach, we tried to understand how the spatial distribution of the complexity-related age-correlation looks like, without prior parcellation into regions or networks. Figure 7 shows that the positively correlated regions are not randomly spread over the whole brain. When visualizing the searchlight results for 99% EV energy, clusters in large frontal inferior and sensorimotor areas become apparent. These regions seem to shrink when we remove more and more Eigenvectors from nMPSE, leaving only some small, concise areas, congruent to previous results (compare Table 1 and Table 2). Also, for only 50% of EV energy, the significant regions are not randomly scattered within the regions reaching significance with 99% of EV energy, but seem to be seed regions to those.

**Figure 8:**
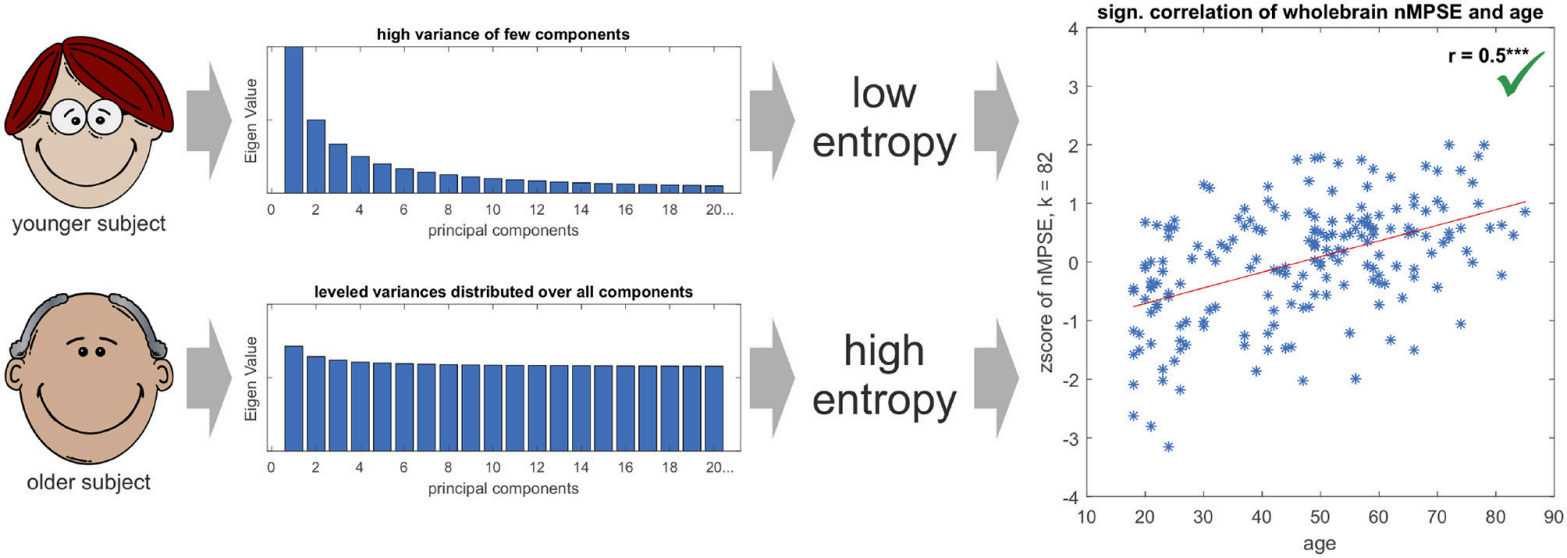
The Eigenvalue spectrum in younger subjects is comprised of few components with high and many components with low Eigenvalues, resp. variances in the direction of the corresponding Eigenvectors. On the contrary, the spectrum for elderly subjects is more leveled and not so much dominated by single components. This leads to younger subjects having a lower Eigenvalue spectrum entropy than older subjects, resulting in a positive significant age - nMPSE correlation (for demonstration purposes EV spectra are displayed in an exaggerated manner, as true differences in EV distributions are subtle).

Finally, the question might arise, why brain entropy is positively correlated with age. Is brain activity in older brains really more complex? Here, we have to take a closer look on the exact meaning of complexity. A signal is less complex (or has a lower entropy) if the signal is dominated by only a few strong components, e.g., sensory networks for younger subjects. If then in older brains other (possibly compensatory) tasks become more demanding, the EV energy would be more equally leveled and thus lead to a higher entropy. From this perspective the signal is more complex. Mathematically spoken, this effect can be explained by a more equally distributed EV spectrum for the elderly subjects, and thus leading to a higher entropy for an increasing *k* in respect to younger subjects (see Figure 8). Another scenario that might have been possible is, that using only the first dimensions leads to high age-complexity correlations and adding more and more dimensions diminishes correlation strength. This would mean that most of the information, relevant for aging, would be encoded in the first dimensions. So, in a way, the characteristic curve of nMPSE describes the distribution of the investigated problem on dimensional subspaces. Especially the intersection point of Ω and nMPSE might be of great value, as it could be used as a biomarker, for instance comparing early and later stages of neurodegenerative diseases.

In summary, the analysis of dimensional complexity of resting-state functional connectivity promises to be a fruitful resource for finding biomarkers. Here, healthy aging served as a proof of concept to provide a method to further study human brain complexity. The intersection point of Ω and nMPSE might be a way to compare the complexity of different problems, e.g., Parkinson's disease progression vs. healthy controls or earlier vs. later stages of Alzheimers disease. Another interesting research question might be the performance of a modified, *k* restricted Ω, compared to nMPSE, as well as a detailed analysis of the properties of dimensions with close to zero Eigenvalues, which are discarded in nMPSE. A windowed approach, looking at temporal dynamics, might reveal different complexity states and help understanding temporal processes in the brain (especially for task experiments). Answering these questions are computationally very demanding and out of the scope of this first publication, but might be very insightful in the future.

## Ethics statement

This study used human subject recordings from an open access data set. The dataset was published with a positive ethics statement. Thus, this article is exempt from an additional ethics committee approval.

## Author contributions

Main and corresponding author is NS. NS performed the analysis and designed the figures. AM was involved in planning and supervised the work. NS and AM drafted the manuscript. EF administered the data preprocessing and principal subspace computations. TM aided in interpreting the results. All authors discussed the results and commented on the manuscript.

### Conflict of interest statement

The authors declare that the research was conducted in the absence of any commercial or financial relationships that could be construed as a potential conflict of interest.

## Footnotes

^1^1000 Functional Connectomes Project datasets are freely accessible at www.nitrc.org/projects/fcon_1000/.

## Supplementary material

The supplementary material provides further figures and tables for intermediate nMPSE results at different levels of k. Also a Matlab implementation for nMPSE is provided.
