# Supplementary material for: Dimensional Complexity of the Resting Brain in Healthy Aging, Using a Normalized MPSE
Source: Front Hum Neurosci. 2018 Nov 19;12:451. doi: 10.3389/fnhum.2018.00451 (PMC6252312; doi:10.3389/fnhum.2018.00451)
Supplement: Supplementary file 1 [file Data_Sheet_1.ZIP › Data Sheet 1/SupplementaryMaterial.pdf]

SUPPLEMENTARY MATERIAL

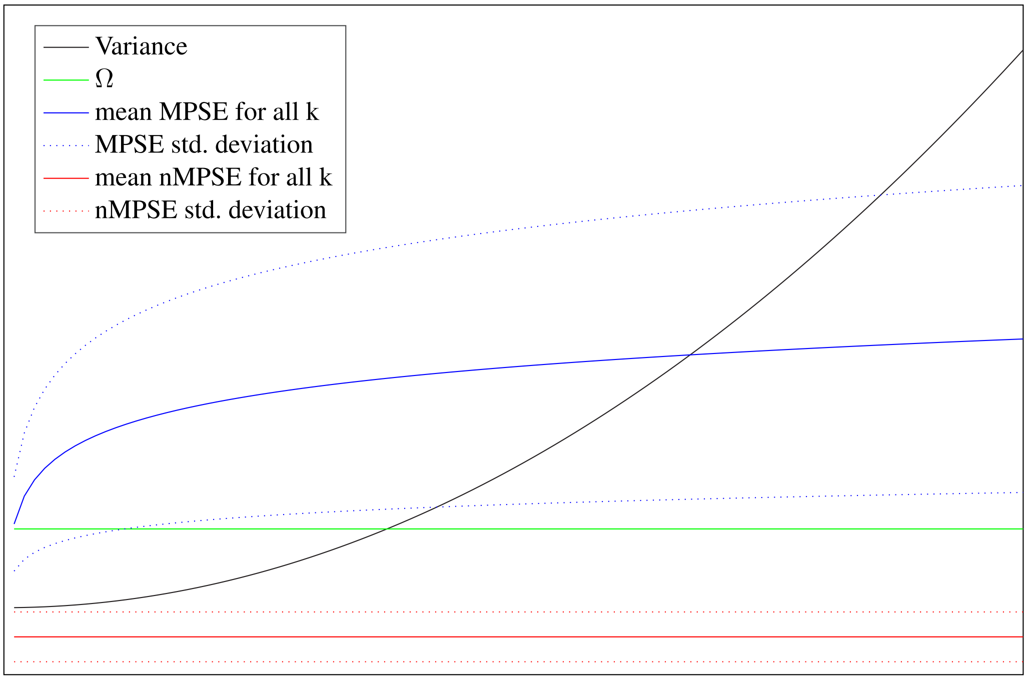

Figure 9. Random data (5000 dimensions over 120 time steps) with increasing variance: Ω stays constant while MPSE increases for every k, nMPSE in the contrary stays constant for every k.

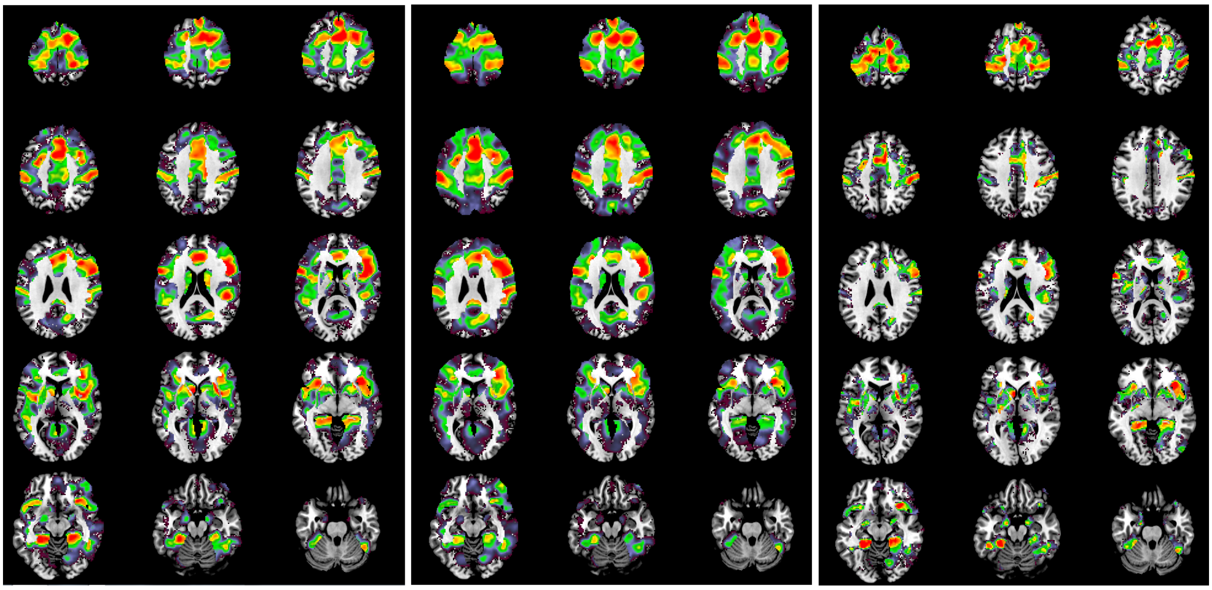

Figure 10. Searchlights: significant voxels for Ω, nMPSE ($k = 29$, 35% of EV-Energy) and nMPSE ($k = 6$, 7% of EV-Energy)

Table 3. Minimal percentage of EV energy (resp. dimensions) needed for a significant age differentiation within each AAL region using nMPSE. Asterisks denote that this region also reaches significance using Ω.

AAL Area	minimal k	EV energy	AAL Area	minimal k	EV energy
Frontal Med Orb L	2	29%	Frontal Inf Tri L	12	64%
Insula R	2	29%	Calcarine R	12	64%
ParaHippocampal L	2	29%	Precuneus R	12	64%
ParaHippocampal R	2	29%	Pallidum L	12	64%
Amygdala L	2	29%	Precentral L	13	66%
Amygdala R	2	29%	Parietal Sup L	13	66%
Lingual R	2	29%	Parietal Inf L	13	66%
Fusiform R	2	29%	Cingulum Post R	14	68%
Caudate L	2	29%	Cuneus L	14	68%
Caudate R	2	29%	Frontal Mid Orb L	15	69%
Putamen R	2	29%	Olfactory L	15	69%
Thalamus L	2	29%	SupraMarginal R	15	69%
Heschl L	2	29%	Frontal Sup R	17	72%
Temporal Pole Sup L	2	29%	Frontal Sup Medial R	17	72%
Frontal Inf Orb R	3	36%	Lingual L	17	72%
Insula L	3	36%	Frontal Sup L	18	73%
Cingulum Ant L	3	36%	Occipital Sup L	18	73%
Paracentral Lobule L	3	36%	Frontal Mid R	19	74%
Paracentral Lobule R	3	36%	Cuneus R	19	74%
Putamen L	3	36%	Frontal Mid L	21	76%
Frontal Inf Oper R	4	41%	Frontal Sup Medial L	21	76%
Frontal Inf Tri R	4	41%	Parietal Inf R	21	76%
Supp Motor Area R	4	41%	Temporal Mid R	21	76%
Precentral R	5	46%	Frontal Sup Orb R	26	80%
Frontal Inf Oper L	5	46%	Calcarine L	26	80%
Supp Motor Area L	5	46%	Temporal Sup R	29	82%
Cingulum Ant R	5	46%	Temporal Mid L	29	82%
Thalamus R	5	46%	Occipital Sup R	30	83%
Heschl R	5	46%	Parietal Sup R	32	84%
Temporal Inf R	5	46%	Occipital Mid L	42	89%
Cingulum Post L	6	50%	Temporal Inf L	51	93%
Pallidum R	6	50%	Occipital Mid R	56	95%
Fusiform L	7	53%	Angular L	56	95%
Postcentral R	7	53%	Angular R	74	99%
Temporal Sup L	7	53%			
Rolandic Oper R	8	56%	Frontal Sup Orb L	n.s.	
Cingulum Mid L	8	56%	Olfactory R	n.s.	
Cingulum Mid R	8	56%	Rectus L	n.s.	
Hippocampus L	8	56%	Rectus R	n.s.	
Postcentral L	8	56%	Hippocampus R	n.s.	
Frontal Inf Orb L	9	58%	Occipital Inf L	n.s.	
Rolandic Oper L	9	58%	Occipital Inf R	n.s.	
Temporal Pole Sup R	9	58%	Temporal Pole Mid L	n.s.	
Frontal Mid Orb R	11	63%	Temporal Pole Mid R	n.s.	
Frontal Med Orb R	11	63%			
SupraMarginal L	11	63%			
Precuneus L	11	63%			

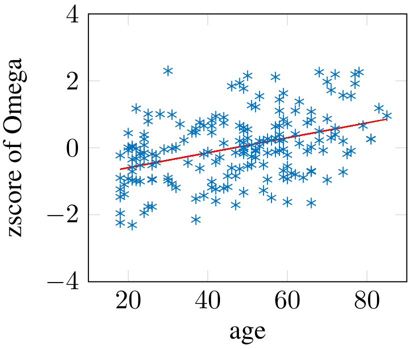

(11a) Whole brain, r = 0.39936

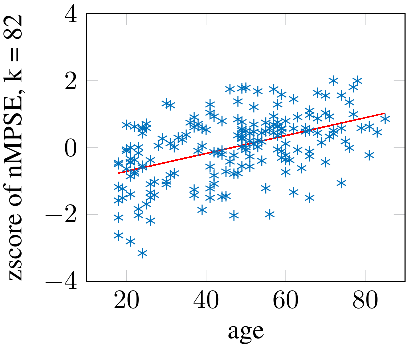

(11b) Whole brain, r = 0.47796

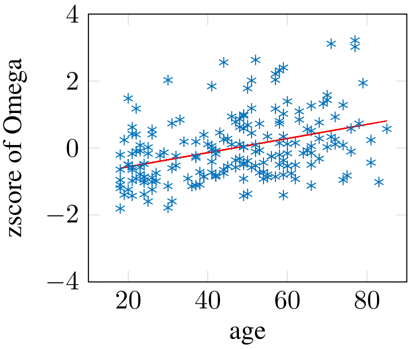

(11c) RSN Posterior Salience, r = 0.37797

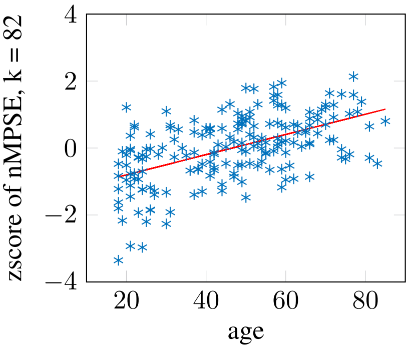

(11d) RSN Posterior Salience, r = 0.54025

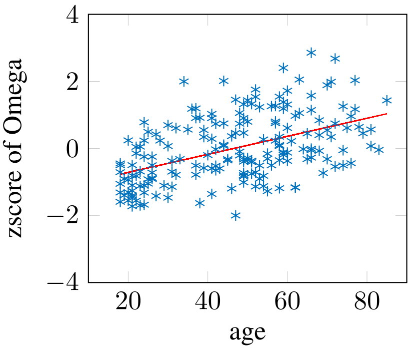

(11e) AAL Frontal Inf. Operculum, r = 0.48153

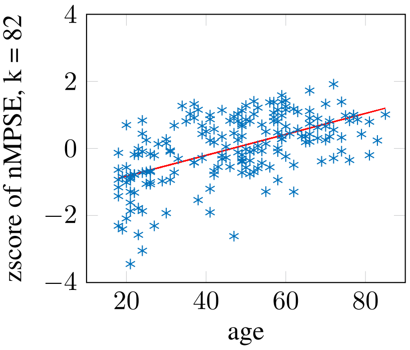

(11f) AAL Frontal Inf. Operculum, r = 0.55929

Figure 11. Age correlation of Omega vs. nMPSE

Table 4. Top 10 significant voxels for Ω Search Lights

X	Y	Z	r	AAL Region
46	18	16	0.5764	Frontal Inf Oper R
10	8	56	0.56563	Supp Motor Area R
36	24	-8	0.55143	Frontal Inf Orb R
48	-28	24	0.54946	SupraMarginal R
-60	10	18	0.53517	Frontal Inf Oper L
38	34	6	0.53437	Frontal Inf Tri R
-6	8	0	0.53345	Caudate L
4	32	22	0.53221	Cingulum Ant R
8	28	46	0.53173	Frontal Sup Medial R
-4	6	46	0.52814	Supp Motor Area L

Table 5. Top 10 significant voxels for nMPSE ($k = 14$, 17% of EV-energy) Search Lights

X	Y	Z	r	AAL Region
46	18	16	0.5932	Frontal Inf Oper R
10	6	56	0.5925	Supp Motor Area R
-60	10	18	0.5458	Frontal Inf Oper L
36	26	-8	0.5431	Frontal Inf Orb R
50	-22	26	0.5395	SupraMarginal R
52	-28	48	0.5325	Postcentral R
10	34	30	0.5239	Cingulum Mid R
-60	-24	46	0.5189	Parietal Inf L
-6	10	0	0.514	Caudate L
-24	0	54	0.5103	Frontal Mid L

Table 6. Top 10 significant voxels for nMPSE ($k = 22$, 22% of EV-energy) Search Lights

X	Y	Z	r	AAL Region
46	20	18	0.6265	Frontal Inf Tri R
6	6	52	0.6062	Supp Motor Area R
-62	12	18	0.5721	Frontal Inf Oper L
50	-22	26	0.5593	SupraMarginal R
36	26	-6	0.5564	Insula R
-60	-24	46	0.5517	Parietal Inf L
-18	8	60	0.5369	Frontal Sup L
-30	32	20	0.5326	Frontal Mid L
-8	-38	28	0.5174	Cingulum Post L
-38	-40	58	0.5164	Postcentral L

Table 7. Top 10 significant voxels for nMPSE ($k = 29$, 35% of EV-energy) Search Lights

X	Y	Z	r	AAL Region
48	18	18	0.6406	Frontal Inf Tri R
6	8	52	0.6142	Supp Motor Area R
-62	12	18	0.5843	Frontal Inf Oper L
-58	-28	46	0.5695	Parietal Inf L
52	-22	26	0.5691	SupraMarginal R
36	26	-6	0.5669	Insula R
-18	8	60	0.5482	Frontal Sup L
-30	32	20	0.544	Frontal Mid L
-40	-40	58	0.5295	Postcentral L
-8	-38	28	0.529	Cingulum Post L

Table 8. Top 10 significant voxels for nMPSE ($k = 37$, 45% of EV-energy) Search Lights

X	Y	Z	r	AAL Region
48	18	18	0.6487	Frontal Inf Tri R
6	8	50	0.6209	Supp Motor Area R
-60	12	18	0.5941	Frontal Inf Oper L
-58	-28	46	0.5818	Parietal Inf L
58	-24	40	0.5773	SupraMarginal R
36	26	-6	0.5747	Insula R
-20	6	58	0.5565	Frontal Sup L
-30	32	20	0.5532	Frontal Mid L
-8	-38	28	0.5353	Cingulum Post L
-4	8	2	0.5253	Caudate L

Table 9. Top 10 significant voxels for nMPSE ($k = 45$, 55% of EV-energy) Search Lights

X	Y	Z	r	AAL Region
46	20	18	0.6536	Frontal Inf Tri R
4	6	52	0.6239	Supp Motor Area R
-60	12	18	0.6009	Frontal Inf Oper L
-58	-30	48	0.592	Parietal Inf L
58	-24	40	0.5847	SupraMarginal R
46	-36	58	0.5836	Postcentral R
-20	6	58	0.563	Frontal Sup L
-30	32	20	0.5582	Frontal Mid L
-40	-42	60	0.5455	Postcentral L
-8	-38	28	0.5407	Cingulum Post L

Table 10. Top 10 significant voxels for nMPSE ($k = 53$, 65% of EV-energy) Search Lights

X	Y	Z	r	AAL Region
46	20	18	0.6564	Frontal Inf Tri R
6	8	52	0.6255	Supp Motor Area R
28	4	56	0.6088	Frontal Mid R
-60	12	16	0.6067	Frontal Inf Oper L
-58	-32	48	0.6002	Parietal Inf L
46	-36	58	0.59	Postcentral R
58	-24	40	0.5892	SupraMarginal R
-20	6	58	0.5669	Frontal Sup L
-30	32	20	0.5585	Frontal Mid L
-40	-42	60	0.5506	Postcentral L

Table 11. Top 10 significant voxels for nMPSE ($k = 61$, 74% of EV-energy) Search Lights

X	Y	Z	r	AAL Region
48	18	18	0.6596	Frontal Inf Tri R
4	6	52	0.6274	Supp Motor Area R
28	4	56	0.6116	Frontal Mid R
-60	12	18	0.6103	Frontal Inf Oper L
-58	-32	48	0.6074	Parietal Inf L
46	-36	58	0.5949	Postcentral R
60	-24	40	0.5925	SupraMarginal R
-20	8	58	0.5707	Frontal Sup L
-30	32	20	0.5602	Frontal Mid L
-8	-38	28	0.5548	Cingulum Post L

Table 12. Top 10 significant voxels for nMPSE ($k = 68$, 83% of EV-energy) Search Lights

X	Y	Z	r	AAL Region
48	18	18	0.663	Frontal Inf Tri R
4	6	52	0.6281	Supp Motor Area R
28	6	56	0.6137	Frontal Mid R
-58	-32	48	0.6133	Parietal Inf L
-60	12	18	0.6102	Frontal Inf Oper L
46	-36	58	0.5981	Postcentral R
60	-24	40	0.5955	SupraMarginal R
-20	8	58	0.5736	Frontal Sup L
-32	30	22	0.5624	Frontal Mid L
-8	-38	28	0.5604	Cingulum Post L
